# Neuroscience meets the classroom: a scoping review of translational research in educational neuroscience (2015–2025)

**DOI:** 10.3389/fpsyg.2026.1853717

**Published:** 2026-07-07

**Authors:** Chiara Fante, Flavio Manganello

**Affiliations:** Institute for Educational Technology (ITD), National Research Council (CNR), Genova, Italy

**Keywords:** co-design, educational neuroscience, knowledge translation, neuroscience-informed teaching, researcher-educator collaboration, school-based implementation, scoping review, translational research

## Abstract

Translating findings from educational neuroscience (EN) into teaching practice remains a complex task, particularly in authentic school contexts. This scoping review examines how EN research has engaged with real-world school settings between 2015 and 2025, focusing on theoretical foundations, translational processes, researcher-educator collaboration, participant characteristics, and conditions supporting knowledge translation. Following PRISMA-ScR reporting standards and JBI scoping review guidance, searches were conducted in Scopus, Web of Science, and ERIC, complemented by backward reference list searching. Twenty-four empirical studies met the predefined inclusion criteria. Data were charted across six domains and synthesized through a narrative approach, with methodological quality appraised using the Mixed Methods Appraisal Tool. The synthesis was structured through a multidirectional T1-T4 translational framework, ranging from the development of teaching applications based on basic research to their systemic dissemination within educational policy and practice. School-based EN research is expanding but remains conceptually and methodologically heterogeneous. Most studies occupied hybrid T2-T3 positions, combining the evaluation of EN-informed applications with implementation in school practice, while T4-level translation was not represented as a primary objective. Theoretical approaches were mainly grounded in cognitive neuroscience, especially neuroplasticity, executive functions, and attention, but were often integrated with psychological and educational frameworks. Thirteen studies drew directly on primary neuroscientific evidence, whereas 11 relied mainly on synthesized EN models. Co-design and co-investigation were frequent and tended to co-occur with clearer translational pathways, although collaboration varied in depth and reciprocity. Participant characteristics were inconsistently used to inform design. Structural constraints, limited teacher preparation, conceptual gaps, and weak fidelity monitoring emerged as recurrent barriers, while institutional support, curriculum alignment, conceptual mediation, and iterative feedback loops facilitated translation. Advancing school-based EN will require stronger theoretical transparency, more systematic collaboration reporting, greater attention to learner and school contexts, and clearer pathways toward systemic uptake.

## 1 Introduction

Over the past two decades, educational neuroscience (EN) has emerged as an interdisciplinary field at the intersection of cognitive neuroscience, developmental psychology, and the learning sciences, proceeding from the premise that evidence about how the brain learns can inform how teaching is designed and delivered (Christodoulou and Gaab, 2009; Fischer et al., 2010; Goswami, 2004; Han et al., 2019). More recent contributions continue to refine this translational agenda, highlighting the evolving nature of the researcher-practitioner dialog (Thomas and Arslan, 2025; Tokuhama-Espinosa and Nouri, 2023). This premise is theoretically productive but practically demanding. Pedagogy, cognitive psychology, and the neuroscience of learning developed largely in parallel for over a century, and their convergence in EN has not yet completely resolved the conceptual distance between laboratory findings and classroom practice (Morris and Sah, 2016; Thomas et al., 2019).

Learning involves multiple interacting cognitive and neural systems whose instructional implications are not easily disentangled; moreover, teaching environments are contextually complex, and the heterogeneity of learners is one of several features of authentic classrooms that resist the controlled conditions under which neuroscientific findings are typically generated. These challenges reflect the structural nature of a gap between two epistemological traditions that EN is still working to bridge.

A boundary distinction central to this review concerns brain-based learning (BBL). The label covers a heterogeneous body of work, ranging from interventions whose design is demonstrably grounded in peer-reviewed neuroscientific evidence to commercialized BBL programs that rely on popularized or generic references to brain function and have been criticized for simplified or poorly validated applications of neuroscientific knowledge (Edelenbosch et al., 2015; Howard-Jones, 2007). Neuroscientific terminology can lend perceived credibility to claims regardless of their actual explanatory value (Weisberg et al., 2008; Legrenzi and Umiltà, 2011). EN seeks to provide a framework through which neuroscientific knowledge can enrich educational practice in ecological contexts (Ansari and Coch, 2006; Ansari et al., 2012; Feiler and Stabio, 2018; Han et al., 2019), a distinction that remains contested (Howard-Jones and Holmes, 2017; Szűcs and Goswami, 2007) but that guides the eligibility criteria applied in this review.

### 1.1 Translational frameworks in educational neuroscience

Scholars increasingly argue that the relationship between neuroscientific evidence and educational practice cannot be conceived as a unidirectional transfer from laboratory to classroom (Donoghue and Horvath, 2016; Tokuhama-Espinosa, 2010; Tommerdahl, 2010). In fact, the pedagogical relevance of neuroscientific findings is not determined in controlled experimental settings but emerges through interpretative processes that take place in real educational contexts, in collaboration with teachers’ professional expertise and the institutional context of schools. From this transactional perspective, EN is a collaborative endeavor in which contextual factors, professional expertise, and institutional conditions actively shape how neuroscientific knowledge is interpreted and put into practice (Tokuhama-Espinosa, 2010).

Translational frameworks offer one principled way to conceptualize this process, and their application to EN has a traceable disciplinary genealogy. The foundational model was proposed in genomic medicine by Khoury et al. (2007), who articulated four phases of translation research organized around the development of evidence-based guidelines: moving a basic discovery toward a candidate health application (T1); assessing the value of that application for health practice (T2); transferring evidence-based guidelines into health practice through dissemination, implementation, and diffusion research (T3); and evaluating the real-world health impact of the application at population level (T4). Critically, Khoury et al. (2007) emphasized that these phases are not strictly sequential, with research overlapping across stages and generating feedback loops that frame translation as an iterative, multidirectional process rather than a simple pipeline. Keramaris et al. (2008) extended this argument by characterizing translation as a dialog between scientific priorities and the needs of society, health systems, and institutional contexts. Within EN, the framework was first applied to specific learning disorders by Dresler et al. (2018), who proposed a multidirectional model connecting neuroimaging findings to mechanisms, diagnosis, intervention, and community practice, with information flowing in either direction, while acknowledging that the community and education component remained the most consistently underdeveloped. Building on this trajectory, Fante (2024) proposed the TRAIN framework (*Translating Research into Active Instruction*), conceptualizing the application of neuroscientific evidence in education throughout a four-stage continuum, ranging from the development of teaching applications based on basic research (T1), to their evaluation and synthesis into evidence-based guidelines (T2), to their implementation in practice (T3) and to their dissemination within educational systems and policies (T4).

The present review draws on the T1-T4 translational framework as traced from Khoury et al. (2007) in genomic medicine and Keramaris et al. (2008), as adapted to educational neuroscience by Dresler et al. (2018), and as operationalized for school contexts through the TRAIN framework (Fante, 2024). The framework is treated here as multidirectional and non-sequential rather than as a linear pipeline, in line with its formulation in the cited sources. It is used in two respects: as the conceptual basis for focusing on authentic school settings as the primary site of EN knowledge translation, and as a source of analytical categories for characterizing translational processes across the included studies.

### 1.2 Rationale

Despite growing conceptual elaboration and increasing recognition of the importance of researcher-practitioner collaboration (Coch and Daniel, 2025), available evidence has not been systematically mapped. Thomas et al. (2019) survey EN broadly across methodological, conceptual, and disciplinary dimensions, including discussion of cognitive constructs, neuroimaging methods, and the wider science-policy interface; they do not, however, examine how EN principles are operationalized in empirical research in authentic school settings, nor do they document researcher-educator collaboration or participant characteristics. The present scoping review is narrower and complementary in scope: it focuses specifically on empirical translation in authentic school contexts, structures the synthesis around the T1–T4 translational framework, and foregrounds researcher-educator collaboration and the conditions supporting knowledge translation into classroom practice. Adjacent scoping reviews, such as Privitera’s (2021) examination of neuroscience training for teachers, have addressed specific intervention types rather than the full range of school-based EN research and its translational foundations.

To the authors’ knowledge, no review has previously mapped the theoretical grounding, translational processes, and participant characteristics of school-based EN studies across a defined period. Three gaps stand out: the theoretical foundations informing this research remain uncharted at the field level; researcher-educator collaboration is poorly documented despite being widely considered essential to meaningful translation; and participant characteristics are inconsistently reported, limiting inferences about the learner populations represented in the evidence base.

The present scoping review addresses these gaps as follows. The first gap, on the uncharted theoretical foundations of school-based EN research, is engaged by RQ1, which examines the theoretical principles informing the corpus and the relationship between primary neuroscientific evidence and synthesized EN frameworks. The second gap, on the under documentation of researcher-educator collaboration, is engaged by RQ2, which examines translational processes and the forms of collaboration documented across the corpus. The third gap, on inconsistent reporting of participant characteristics, is engaged by RQ3, which examines how participant characteristics are reported and whether they shape research design. RQ4 cross-cuts the three gaps by examining the conditions associated with knowledge of translation into educational practice. The four research questions are stated formally in Section 1.3.

### 1.3 Objectives

A scoping review was the most appropriate design, given the field’s heterogeneous theoretical frameworks, diverse study designs, and inconsistent terminological conventions that preclude meaningful meta-analytic synthesis (Arksey and O’Malley, 2005; Peters et al., 2020; Tricco et al., 2018). Where evidence is sparse or inconsistently reported, those gaps are identified as areas warranting further investigation. Consistent with the Joanna Briggs Institute (JBI) methodological guidance for scoping reviews (Peters et al., 2020; Pollock et al., 2023), review questions were formulated using the Population, Concept, Context (PCC) framework. The temporal boundary of 2015–2025 captures a decade of EN research following the consolidation of the field’s translational agenda and allows for tractable yet comprehensive mapping of the evidence. Table 1 summarizes the PCC elements as applied in this study.

**Table 1:** PCC framework table for the scoping review.

Component	Description
Population	Students and/or educators across school levels, from early childhood to secondary education
Concept	Translation of EN principles into school-based educational practices and interventions, including BBL studies whose intervention design is demonstrably grounded in peer-reviewed neuroscientific evidence or established EN frameworks; commercialized BBL programs relying on popularized or generic references to brain function are excluded
Context	Formal school settings; studies published between 2015 and 2025, reflecting a decade of EN research following the consolidation of the field’s translational agenda

The review questions guiding this scoping review are:

*RQ1*: Which theoretical principles guide school-based EN studies?

*RQ2*: How are translational processes and researcher-educator collaborations documented in the literature?

*RQ3*: How are participant characteristics reported in relation to research design across school-based EN studies?

*RQ4*: What conditions are documented in empirical studies as associated with the translation of neuroscientific knowledge into educational practice in school settings?

## 2 Methods

This scoping review was conducted in accordance with the PRISMA-ScR guidelines (Tricco et al., 2018) and the JBI methodological guidance for scoping reviews (Peters et al., 2020; Pollock et al., 2023). The review protocol was registered on the Open Science Framework (OSF) prior to data collection and analysis and is accessible at: https://doi.org/10.17605/OSF.IO/6J4DK.

### 2.1 Eligibility criteria

#### 2.1.1 Inclusion criteria

Studies were eligible if they met all of the following conditions: (1) were explicitly grounded in Educational Neuroscience, Mind, Brain, and Education, neuroeducation, neuroscience-informed education, or an equivalent translational framework connecting neuroscientific evidence with educational practice, with clear reference to specific neuroscience constructs, mechanisms, or findings; (2) involved students at any school level from early childhood through secondary education and/or teachers as participants; (3) were conducted in formal school settings; (4) documented the implementation of an educational action, practice, or intervention informed by neuroscientific knowledge in an authentic school context; (5) were published in English; and (6) appeared between January 2015 and December 2025.

Criterion (1) required explicit connections to peer-reviewed neuroscientific evidence within an EN translational frame. BBL studies qualified only where grounding was demonstrable through primary neuroscience sources or established EN frameworks; commercialized BBL programs, defined as those relying on popularized or generic references to brain function, did not meet this threshold and fell under exclusion criterion (5). Criterion (4) required that neuroscientific knowledge actively informed the design or delivery of an educational action; teacher professional development studies qualified only where subsequent implementation of EN-informed practices in classroom contexts was documented.

The 2015–2025 timeframe was selected for two convergent reasons. The lower bound corresponds to the post-2014 consolidation of the EN field around translational concerns, following Howard-Jones (2014) on neuromyths and culminating in Dresler et al. (2018), the framework benchmark adopted here. The decade window is consistent with scoping review practice for rapidly evolving interdisciplinary fields and with prior EN syntheses (Thomas et al., 2019). The upper bound corresponds to the search execution date.

#### 2.1.2 Exclusion criteria

Studies were excluded if they met any of the following conditions: (1) not a primary empirical publication, including reviews of all types, bibliometric analyses, theoretical papers, opinion pieces, commentaries, editorials, book chapters, dissertations, theses, and non-peer-reviewed conference abstracts; (2) published outside January 2015 to December 2025; (3) conducted exclusively in higher education, clinical settings, or informal learning environments without a school-based component; (4) did not involve students at any school level from early childhood through secondary education, or teachers in formal school settings; (5) demonstrated insufficient or inappropriate translation of neuroscientific evidence into educational design, delivery, or framing. This criterion excludes: (a) studies citing commercialized BBL principles without reference to primary neuroscience research or established EN theoretical frameworks; (b) studies making claims about brain function or learning that contradict or misrepresent established neuroscientific evidence; (c) purely correlational or observational studies measuring cognitive, neural, or behavioral variables without any EN-informed educational action; (d) teacher professional development studies measuring exclusively knowledge, beliefs, or attitudes about neuroscience or about EN principles, without any documented connection to subsequent classroom implementation; documentation of implementation may take the form of classroom observation, fidelity measurement, student-level outcomes, or teachers’ own accounts of applying EN-informed practices with their students, provided that such accounts refer to specific classroom episodes rather than to general intentions or beliefs; (e) studies citing EN or BBL only as background, without such knowledge informing the design or delivery of the educational action; (f) studies in which neuroscientific knowledge constitutes the subject matter taught to participants, such as neuroscience literacy or brain awareness curricula delivered to students or teachers, rather than informing the design of an educational action; (g) studies investigating cognitive, behavioral, or developmental phenomena with possible neuroscientific relevance, such as executive functions, working memory, inhibitory control, attention, or cognitive flexibility, without explicitly framing the educational action as an instance of EN, Mind, Brain, and Education, neuroeducation, neuroscience-informed education, or BBL grounded in neuroscientific evidence. This sub-criterion also applies to school-based interventions derived from cognitive psychology or cognitive neuroscience where the translational connection to EN was indirect, implicit, or inferred by the reviewers rather than stated by the authors; (6) full text not retrievable after documented retrieval procedures, including institutional access, interlibrary loan requests.

### 2.2 Information sources and search strategy

A systematic search was conducted across three bibliographic databases: Scopus, Web of Science (Core Collection), and ERIC, selected for their complementary coverage of educational, psychological, and neuroscientific literature. The search was restricted to studies published in English between 2015 and 2025. On ERIC, the peer-reviewed filter was applied directly through the database interface.

The search strategy was built around two conceptual clusters combined with the Boolean operator AND. The first targeted the theoretical domain of EN through the terms: “educational neuroscience,” neuroeducation* (with explicit forms neuroeducation, neuro-education, neuroeducational on ERIC, where wildcard support is more limited), “mind, brain, and education,” and “mind brain education.” The second anchored the search to formal school contexts through: school*, classroom*, preschool*, and kindergarten*. The full search strings, as adapted for each database, are reported in Table 2. The term “science of learning” was deliberately excluded from the Concept block. Although the science of learning shares conceptual ground with EN, recent scoping work by Privitera et al. (2023) clarifies that the two are distinct enterprises, with the science of learning explicitly framed as not including reference to the design of learning experiences, environments, or programs, nor to the practice of teaching. Because the present review is concerned precisely with the translation of neuroscientific evidence into authentic school practice, retaining “science of learning” as a self-identifier would have diluted the corpus with studies disciplinarily positioned outside this translational focus.

**Table 2:** Databases and search strings used.

Database	Search string used
Scopus	(TITLE-ABS-KEY (“educational neuroscience” OR neuroeducation* OR “neuro-education” OR “mind, brain, and education” OR “mind brain education”) AND TITLE-ABS-KEY(school* OR classroom* OR preschool* OR kindergarten*)) AND PUBYEAR > 2014 AND PUBYEAR < 2026 AND (LIMIT-TO(LANGUAGE, “English”))
Web of science	TS = (“educational neuroscience” OR neuroeducation* OR “neuro-education” OR “mind, brain, and education” OR “mind brain education”) AND TS = (school* OR classroom* OR preschool* OR kindergarten*) AND PY = (2015–2025) AND LA = (English)
ERIC	(“educational neuroscience” OR neuroeducation OR “neuro-education” OR neuroeducational OR “mind, brain, and education” OR “mind brain education”) AND (school* OR classroom* OR preschool* OR kindergarten*) pubyearmin:2015 pubyearmax:2025
	Filter: peer reviewed only

No methodological or document type filters were applied at the database level, as imposing such restrictions risked excluding empirical studies indexed under non-standard document types; eligibility was assessed during screening instead. Following full-text screening, reference lists of all included studies were examined for additional sources in accordance with JBI guidance for backward reference list searching (Peters et al., 2020); identified records were assessed against the same criteria and documented in the PRISMA-ScR flow diagram. The database searches were conducted in May 2026.

### 2.3 Screening and selection of sources of evidence

All de-duplicated records were uploaded to a shared Rayyan project with bibliographic information visible throughout. At Stage 1, both inclusion and exclusion criteria were applied to titles and abstracts. Records lacking sufficient detail to confirm theoretical grounding or the translational dimension were advanced to Stage 2 rather than excluded, ensuring that studies not clearly identifiable as eligible at the abstract level were not prematurely removed.

A calibration exercise was conducted in which both reviewers independently screened 43 records (approximately 10% of the de-duplicated corpus), yielding Cohen’s *κ* = 0.807 (95% CI: 0.551–1.000), exceeding the pre-specified threshold of ≥0.80 and superseding preliminary estimates in the registered protocol. Disagreements clustered around three patterns: divergent interpretations of the concept criterion; uncertainty about teacher participant studies where school-based implementation was implied but not directly measured; and difficulty distinguishing empirically grounded model development from purely theoretical papers. All discrepancies were resolved through structured discussion, and screener instructions were revised to provide explicit decision rules for each case. The remaining 394 records were divided equally between the two reviewers. Records that clearly met the exclusion criteria were screened by a single reviewer and were not systematically cross-checked by the second reviewer. This represents a deviation from the JBI recommendation that all records undergo independent dual screening (Peters et al., 2020), a decision made on practical grounds given the size of the corpus and available resources. The associated risk of bias was mitigated by the high calibration agreement, the explicit decision rules incorporated into the screener instructions, and the conservative advancement policy whereby records of uncertain eligibility were advanced to Stage 2 rather than excluded.

At Stage 2, all six exclusion criteria defined in Section 2.1.2 were applied comprehensively to full texts, with particular attention to the seven sub-criteria of Criterion 5. Criteria 1, 2, 3, 4, and 6 were verified through publication metadata, study setting, participant characteristics, and retrieval records. Criterion 5 required closer textual scrutiny: for each full text, screeners traced the chain from cited neuroscientific sources to the design or delivery of the educational action. Three configurations warranted particular care: whether neuroscientific knowledge oriented educational design rather than constituting the subject matter of instruction (Criterion 5(f)); whether the educational action was explicitly framed by the authors as an instance of EN, Mind Brain and Education (MBE), neuroeducation, neuroscience-informed education, or BBL grounded in neuroscientific evidence, rather than within cognitive psychology or cognitive neuroscience without an explicit EN translational anchor (Criterion 5(g)); and, for teacher professional development studies, whether the paper documented a connection to subsequent classroom implementation through observation, fidelity measurement, student-level outcomes, or teachers’ accounts referring to specific classroom episodes rather than general intentions or beliefs (Criterion 5(d)).

### 2.4 Data charting process and data items

Data charting was conducted using a structured form developed by the authors, informed by the PCC framework and the JBI Manual for Evidence Synthesis (Aromataris et al., 2024), and organized around six thematic domains: study characteristics; theoretical foundations; translational processes; researcher-educator collaboration; participant-related design elements; and outcomes and process data, the last covering barriers, facilitators, and implementation strategies.

Regarding translational processes, studies were deductively assigned to the T1–T4 stages based on the structural alignment between their research design and the operational objectives defined in the TRAIN framework (Fante, 2024). Specifically:

- T1 (*discovery to educational application*): identified in studies focused on translating basic neuroscientific findings into new pedagogical prototypes or materials;
- T2 (*educational application to evidence-based guideline*): assigned to studies that employ pre-test/post-test or quasi-experimental designs to test the effectiveness of these applications in authentic settings;
- T3 (*guidelines to educational practices*): used for studies that examine dissemination, implementation, and factors affecting the adoption of established evidence-based guidelines;
- T4 (*practice to educational systems*): reserved for research evaluating systemic impact on policies or institutional frameworks;
- Borderline cases: such as studies involving both the iterative development of a tool and its immediate field testing- were assigned to dual categories (e.g., T2-T3) to reflect their hybrid translational nature and maintain analytical precision.

In addition, collaboration categories (implementation only, consultation, co-design, co-investigation) were operationalized based on the level of involvement of school practitioners (e.g., teachers, educators, instructors, tutors) in relation to researchers and assigned using explicit criteria derived from study descriptions: *implementation only* refers to delivery without involvement in design or evaluation; *consultation* to provision of feedback without participation in decision-making; *co-design* to joint development or adaptation of tools and interventions; and *co-investigation* to partnership across multiple research phases (e.g., design, implementation, data collection, interpretation). Each study was classified according to the highest level of practitioner involvement reported.

Given that the T1–T4 categories derive from a framework independently established in medicine and subsequently adapted to EN across a sequence of applications, their use as analytical lenses does not presuppose the conclusions of the review. Where the data extracted from included studies do not map cleanly onto these categories, that tension is treated as a finding that requires interpretation rather than problems to be resolved through forced classification.

The complete charting matrix is archived in the OSF project. Variables were operationally defined prior to extraction; ambiguities were resolved through discussion and documented in the project log. Each reviewer charted their assigned studies; completed matrices were cross-checked by the other author, with discrepancies resolved through discussion.

### 2.5 Synthesis of results

The synthesis follows the four-phase narrative procedure outlined by Popay et al. (2006). The first phase produced a preliminary synthesis by tabulating charted data across the six thematic domains. The second explored relationships within and between studies, attending to convergences and divergences in theoretical frameworks, collaboration arrangements, and participant characteristics. The third assessed the robustness of emerging patterns against Mixed Methods Appraisal Tool (MMAT) appraisal scores (Hong et al., 2018), flagging findings resting predominantly on methodologically weaker studies. The fourth organized synthesis findings in direct correspondence with the four review questions, supported by frequency counts and descriptive tabulations where appropriate (Peters et al., 2020). Studies not mapping cleanly onto a single T1–T4 category were assigned to the closest level with a qualifying notation; those in which neuroscientific knowledge functioned as background framing were documented as occupying a background-level translational position, treated as a distinct sub-type rather than a formal T1-T4 stage.

Narrative synthesis was selected as the integration approach because it accommodates evidence from quantitative, qualitative, and mixed methods designs without requiring methodological homogenization. The choice carries implications for interpretation that we make explicit here: effect estimates and inferential weight cannot be directly compared across designs, and integration is descriptive and thematic rather than aggregative. The MMAT was applied to characterize methodological quality within each design family separately, and quality considerations are reported alongside the synthesis (Section 3.3) rather than used to weight the contribution of individual studies.

### 2.6 Critical appraisal of individual sources of evidence

Although not mandatory in scoping reviews, critical appraisal was conducted to enhance the rigor and transparency of the synthesis, consistent with JBI guidance (Aromataris et al., 2024; Tricco et al., 2016; Tricco et al., 2018). The MMAT (Hong et al., 2018) was selected for its capacity to accommodate quantitative, qualitative, and mixed methods designs within a single framework. Both reviewers independently appraised all included studies; discrepancies were resolved through discussion. No studies were excluded on appraisal grounds. Findings were integrated into the third synthesis phase to assess the robustness of emerging patterns and flag interpretive claims resting on a limited or methodologically constrained evidence base.

## 3 Results

### 3.1 Selection of sources of evidence

Database searches across Scopus, Web of Science, and ERIC yielded 630 records in total (Scopus: *n* = 238; Web of Science: *n* = 189; ERIC: *n* = 203). Following automated and manual deduplication, 193 duplicate records were removed, leaving 437 records for title and abstract screening. At this stage, records were excluded for wrong population (*n* = 9), wrong concept (*n* = 252), wrong context (*n* = 41), wrong publication type (*n* = 84; non-empirical formats including editorials, commentaries, and conference abstracts without full methods), and wrong year (*n* = 1). The remaining 50 reports were sought for full-text retrieval. All reports were retrieved. Full-text assessment identified a further 30 ineligible records, excluded on the basis of wrong concept (*n* = 15), wrong publication type (*n* = 10), wrong context (*n* = 4), and wrong population (*n* = 1). Twenty studies from the primary database searches were consequently retained for inclusion.

Backward reference list searching was conducted on the 20 studies included from the primary database corpus. Reference lists yielded 521 records; following removal of 316 records outside the 2015–2025 window, 205 proceeded to title and abstract screening. Of these, 180 were excluded (wrong population: *n* = 5; wrong concept: *n* = 63; wrong context: *n* = 18; wrong publication type: *n* = 94), leaving 25 for full-text assessment. Two reports could not be retrieved despite institutional access and interlibrary loan request, and were recorded as not retrieved. A further 18 were excluded (wrong concept: *n* = 16; wrong context: *n* = 1; wrong publication type: *n* = 1). Five met all inclusion criteria. One study (Runganurak et al., 2022) was identified independently in both the primary database corpus and the reference list corpus and was therefore counted only once, yielding a final corpus of 24 included studies.

**Figure 1:**
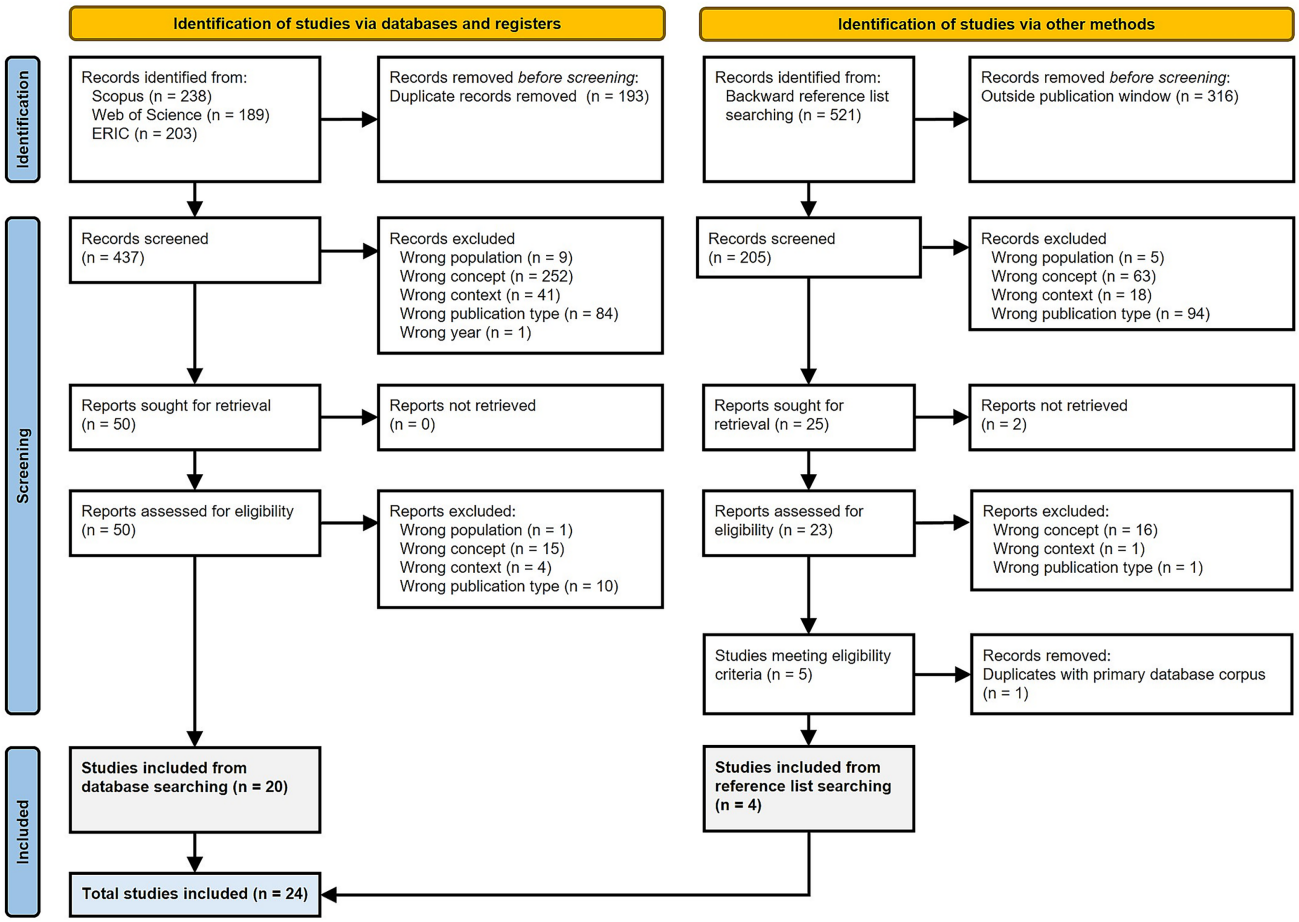
PRISMA flow diagram outlining the screening process, including reasons for exclusions and final sources of evidence.

The combined selection process yielded 24 sources of evidence. The complete search documentation, including the executed search strings, raw database exports, de-duplicated corpus, and reference list file, is archived in the associated OSF project^1^. All screening decisions for both stages and both pathways, with reasons for exclusion, are archived in the associated OSF project^2^. The PRISMA-ScR flow diagram (Figure 1) summarizes screening decisions and reasons for exclusion at each stage (Tricco et al., 2018).

### 3.2 Characteristics of sources of evidence

The 24 included studies span the full temporal window of the review, from 2015 to 2025. Publication was concentrated in the second half of the decade: six studies were published between 2015 and 2019, while 18 were published between 2020 and 2025, with the years 2020 and 2021 together accounting for 7 of the 24 studies. This distribution suggests a period of accelerating empirical engagement between EN and school practice in the years immediately preceding and following the COVID-19 pandemic. A summary of the relevant charted data is presented in Table 3 (Section 3.4); the complete charting matrix, encompassing all 17 extraction fields, is archived in the associated OSF project^3^.

**Table 3:** Results of individual sources of evidence.

ID	Study	Country	Design	Educational level	Setting	Sample size	Theoretical foundations	Translational process	Collaboration	Key outcomes
1	Allix et al. (2024)	France	Quantitative Non-rand	Secondary (grades 6-7)	Multi-school (2 schools)	87 students	Metacognition; brain plasticity; executive functions; dual process theory	T2-T3: Cogni’Scol metacognitive program co-constructed with teachers	Co-design	Significant improvement in brain knowledge and neuromyth reduction; no academic performance gains
2	Anderson et al. (2018)	United States	Mixed methods	Primary (grade 5)	Multi-school (8 districts)	40 teachers and ~3,596 students	Neuroplasticity; growth mindset; Mathematical Mindset framework	T2-T3: blended PD integrating neuroplasticity to challenge fixed-ability beliefs	Co-investigation	Significant shifts in teacher practice, student mindset, and student achievement
3	Bélanger et al. (2025)	Canada	Qualitative	Preschool and primary	Single-school (1 school)	10 teachers	Neuroeducational principles (repeated activation, active learning)	T3: PLC structure embedding neuroeducational principles into teacher practice	Co-investigation	Facilitating and impeding factors identified; curriculum overload and classroom management as main barriers
4	Brown et al. (2024)	United States	Quantitative non-rand	Early childhood (preschool)	Single-school (1 Head Start site)	76 children	HPA-axis regulation; cortisol; social buffering; self-regulation	T1-T2: EN framework used to generate hypotheses about pedagogical mechanisms moderating cortisol	Consultation	Child-directed activity and teacher interaction quality independently predicted lower cortisol
5	Caballero and Llorent (2022)	Spain	Quantitative Non-rand	Secondary (grades 7-8)	Multi-school (3 schools)	209 students	Six universal neuroeducation principles; executive functions; neuroplasticity	T2-T3: two-phase teacher training translating EN into holistic classroom methodology	Co-design	Significant improvements in reading, mathematics, and empathy in experimental groups
6	Chang et al. (2021)	United States	Mixed Methods	K-12	Multi-school (not specified)	14 teachers (6 observed)	Educational neuroconcepts (ENCs); Mind Brain and Education framework; constructivist professional development; Shulman’s knowledge of students	T2-T3: teachers independently applied ENCs to classroom practice after constructivist PD	Co-investigation	ENCs guided pedagogical thinking, reinforced known practices, and shaped real-time instructional decisions
7	Elouafi et al. (2021)	Morocco	Quantitative non-rand	Secondary (grades 7–12)	Multi-school (3 schools)	239 students	Hebb’s law; analogical reasoning; multisensory learning; self-determination theory	T2-T3: four neuropedagogical methods designed from neuroscience findings on memory and engagement	Co-design	Significant improvements on psycho-pedagogical parameters across all four methods
8	Hachem et al. (2022)	Canada	Qualitative	Secondary	Single-school (1 school)	7 teachers (out of 75 PD recipients)	Neuroplasticity; executive functioning; retrieval practice; adolescent brain development	T3: year-long co-designed Professional Development (PD) translating EN into teaching practice	Co-investigation	Deeper EN knowledge; enhanced practice; stronger relationships; increased student engagement
9	Hermida et al. (2015)	Argentina	Quantitative Non-rand	Early childhood (kindergarten)	Single-school (1 school)	49 children	Executive functions; prefrontal cortex development; poverty and cognitive development	T2-T3: interdisciplinary EF-training curriculum co-designed by neuroscientists and educators	Co-design	Significant academic achievement gains; limited cognitive transfer
10	Howard-Jones et al. (2015)	United Kingdom	Qualitative	Secondary (years 7, 9, 10)	Multi-school (2 schools)	46 students across five cycles	Brain reward system; midbrain dopamine and uncertainty; expectation and reward magnitude; neuroeducational research framework	T2-T3: iterative design-based research developing a games-based teaching app from neuroscience-informed pedagogical principles across five cycles	Co-design	Apparent learning gains across cycles; pedagogical principles co-constructed; web app deployed internationally
11	Khng and Mane (2020)	Singapore	Quantitative RCT	Primary (grade 5)	Single-school (1 school)	45 students	Attentional control theory; EEG frequency bands; neural efficiency	T2-T3: deep breathing intervention validated against neurophysiological measures in school	Implementation only	Significant reduction in respiratory rate; neurophysiological effects detected; no behavioral effects
12	Koller (2024)	Multi-country	Mixed methods	Multiple (grades 3–12)	Multi-school (not specified)	15 teachers and 585 students	Panksepp’s neuroevolutionary theory (SEEKING system); neuroplasticity; growth mindset; emotions and learning	T2: explorer mindset framework connected to EN rationale and integrated into existing curriculum; translational logic partially operationalized	Consultation	Significant gains in empowerment and engagement; largest effects in elementary students
13	Letang et al. (2021)	France	Quantitative RCT	Primary (grades 2–5)	Multi-school (48 teachers across schools)	48 teachers and 1,049 students	Executive function and inhibitory control; developmental neuroscience	T2-T3: IC training activities co-designed with teachers for classroom delivery via citizen science platform	Co-design	Significant improvement in inhibitory control in IC training group
14	Mackey et al. (2017)	United States	Quantitative RCT	Primary (grade 5)	Single-school (1 charter school)	46 students	Brain plasticity; executive functions; cognitive training paradigms	T2-T3: neuroscience-informed game-based curriculum targeting processing speed, working memory, and fluid reasoning	Co-design	Significant cognitive composite gains; no transfer to standardized academic tests
15	Massonnié et al. (2020)	France/United Kingdom	Mixed methods	Primary	Multi-school (6 classes)	113 students	Attentional networks; noise effects on cognition; mindfulness; ecological validity	T2-T3: co-constructed noise-reduction interventions grounded in attention neuroscience	Co-investigation	Sound awareness reduced noise interference and annoyance; no behavioral attention effects
16	Ribau (2020)	Portugal	Quantitative Descriptive	Secondary (grades 8-9)	Single-school (1 school)	50 students	Neuroeducation (background framing); attention; emotion-motivation links	T1 (motivational rationale): EN invoked as theoretical rationale for active laboratory station methodology	Consultation	Improved student engagement and perception; mixed academic outcomes
17	Roche-Cerasi et al. (2021)	Norway	Quantitative non-rand	Primary (grade 5)	Single-school (1 school + traffic center)	61 students	Place cells; cognitive map formation; predictive brain; neuroplasticity	T2-T3: six-activity road safety model derived from EN spatial navigation research	Co-design	Significant improvement in visual attention and fixation in experimental group
18	Runganurak et al. (2022)	Thailand	Quantitative RCT	Secondary (grade 10)	Single-school (1 school)	63 students	Educational neuroscience; multi-sensory plates; prefrontal cortex and top-down attention; design-based learning; executive functions	T2-T3: Design-based learning integrated with EN model (DEN) operationalized as five-phase physics instruction	Implementation only	Significant gains in physics achievement, science process skills, scientific mind, attention, working memory, and stress reduction
19	Skjermo et al. (2022)	Norway	Quantitative non-rand	Primary (grade 5)	Multi-school (3 schools)	61 students	Place cells and cognitive maps; predictive brain; neuroplasticity; attentional networks	T2-T3: VR eye-tracking evaluation of the neuroeducation-based road safety curriculum (companion paper to Roche-Cerasi et al., 2021)	Co-design	Significant between-group differences in number and rate of ROI fixations, favoring the new curriculum
20	Srikoon (2019)	Thailand	Quantitative RCT	Secondary (grade 9)	Single-school (1 school)	77 students	Educational neuroscience; Brain-Targeted Teaching Model; research-based learning	T2: 5P neurocognitive instructional model applied in mathematics	Implementation only	Significant post-test gains in mathematics achievement and mood
21	Srikoon et al. (2024)	Thailand	Quantitative RCT	Secondary (grade 9)	Single-school (1 school)	70 students	Neuroconstructivism; Brain-Targeted Teaching Model	T2: STEMEN model operationalizing EN constructs into seven instructional syntaxes	Implementation only	Significant improvements in mathematical literacy and problem-solving
22	Tan et al. (2019)	Canada	Qualitative	Primary (grades 1–6)	Single-school (not explicitly specified)	8 teachers	Neural network hypothesis; hierarchical relational binding theory; attention and awareness	T2-T3: neuroscience-framed learning study with iterative plan-teach-reflect cycles	Co-investigation	Teachers developed theoretical coherence using neuroscience language; sustained application
23	Tan and Amiel (2022)	Canada	Qualitative	Primary (grades 1–6)	Single-school (same site as Tan et al., 2019)	5 teachers	Neural network hypothesis; hierarchical relational binding theory; attention and awareness	T2-T3: phenomenographic learning study examining teacher engagement with neuroscience analogies	Co-investigation	Three categories of deepening understanding; sustained application at one-year follow-up
24	Zhumabayeva et al. (2025)	Kazakhstan	Quantitative non-rand	Primary (grades 1–4)	Multi-school (2 schools)	624 students	Neurodidactics; brain plasticity; executive functions; Cognitive Load Theory	T2-T3: neurodidactic elective curriculum targeting cognitive-motor integration	Implementation only	Significant gains across verbal intelligence, logical reasoning, and cognitive flexibility

T1–T4 refer to translational stages adapted from Khoury et al. (2007) and Dresler et al. (2018): T1, basic-to-applied; T2, development of evidence-based applications; T3, implementation in practice; T4, dissemination into systems and policy; RCT, randomized controlled trial; Non-rand, non-randomized; PD, professional development; ENCs, educational neuroconcepts; MBE, mind, brain, and education; EF, executive functions; HPA-axis, hypothalamic–pituitary–adrenal axis; PLC, professional learning community; EEG, electroencephalogram; VR, virtual reality; ROI, region of interest; DEN, design-based learning integrated with educational neuroscience; STEMEN, science, technology, engineering, and mathematics + educational neuroscience.

Geographically, the corpus reflects a moderate degree of diversity alongside a clear concentration in high-income, Anglophone or Western European contexts. North America contributed eight studies, with equal representation from the United States and Canada. Europe contributed eight studies, including two from France, and one each from Norway, Spain, Portugal, and a Franco-British collaboration. Asia-Pacific contexts were represented by five studies conducted in Thailand, Singapore, and Kazakhstan. One study was conducted in a middle-income country, Morocco, and one study was multi-country. Argentina contributed one further study. No studies were conducted in Eastern Europe, Sub-Saharan Africa, the Middle East, or Oceania.

In terms of study design, the corpus was methodologically heterogeneous. Eight studies employed quantitative non-randomized designs, typically quasi-experimental with pre-post measurement structures. Six studies were quantitative randomized controlled trials, five were qualitative, four used mixed methods design integrating quantitative and qualitative components, and one was a quantitative descriptive case study. The prevalence of non-randomized and qualitative designs reflects the ecological orientation of the corpus: most studies prioritized authenticity of implementation over experimental control.

Educational levels represented across the corpus ranged from preschool to upper secondary. Ten studies were conducted in primary school settings, nine in secondary school settings, and two in early childhood or preschool contexts. Three studies spanned multiple levels simultaneously. This distribution indicates that primary and secondary education together account for the overwhelming majority of school-based EN research in the corpus, while early childhood education remains comparatively underrepresented.

Participant samples varied substantially in composition and size. Sixteen studies involved students as their primary or exclusive research participants. Five studies focused on teachers or school personnel as participants, examining how EN knowledge was acquired, applied, or perceived in professional development contexts. Three studies involved both students and teachers as principal units of analysis. Sample sizes ranged from five to approximately 3,600 participants, with a median of 62. Nine studies had samples of fewer than 50 participants, 9 had samples between 50 and 199, and 6 had samples of 200 or more. Settings were nearly evenly divided, with 13 studies conducted in a single-school context and 11 across multiple schools. The qualitative studies consistently featured small purposive samples, which is appropriate to their design logic, while the largest samples were found in the blended PD study by Anderson et al. (2018), the citizen science RCT by Letang et al. (2021), and the non-randomized cognitive program by Zhumabayeva et al. (2025).

Researcher-educator collaboration was represented across four of the five coded levels; no study reported a complete absence of contact. Nine studies were coded as co-design, seven as co-investigation, three as consultation, and five as implementation only. The prevalence of co-design and co-investigation indicates that a substantial proportion of school-based EN research in this decade engaged educators as partners rather than solely as subjects, though the depth and reciprocity of these arrangements varied considerably. The distribution is examined in detail in Section 3.5.2.

### 3.3 Critical appraisal within sources of evidence

Critical appraisal was conducted on all 24 included studies using the MMAT version 2018 (Hong et al., 2018). No studies were excluded based on appraisal outcomes. Findings were used to assess the robustness of the narrative synthesis, as described in the Methods section (2.5). The complete per-study, per-criterion MMAT appraisal, including reviewer notes and disagreement resolution, is publicly available on OSF^4^.

Overall methodological quality was heterogeneous across the five design categories described in Section 3.2. Six studies were rated as high quality, three as medium-high, ten as medium, two as low-medium, and three as low quality. Both screening questions were met by all studies, except for Ribau (2020), whose data collection was not clearly aligned to explicitly formulated outcome-level questions. This baseline criterion is necessary but minimal, and its near-universal fulfillment does not in itself reflect the quality of the evidence produced.

Among the six RCTs, methodological quality ranged from high (Srikoon et al., 2024) to low (Srikoon, 2019). Randomization procedures and baseline equivalence were adequately documented in three studies (Khng and Mane, 2020; Mackey et al., 2017; Srikoon et al., 2024); the remaining three provided insufficient methodological detail to confirm allocation integrity. Outcome assessor blinding was the most consistently unaddressed criterion, formally documented only in Mackey et al. (2017). Intervention fidelity could be confirmed in two RCTs (Khng and Mane, 2020; Srikoon et al., 2024) and was uncertain in the remaining four.

Among the eight non-randomized quantitative studies, complete outcome data were reported in five, constituting a relative strength of this subgroup. Confounder control was the critical shared limitation, with six studies receiving a negative rating, reflecting absent randomization, uncontrolled baseline non-equivalence, or failure to account for teacher and contextual variability. Hermida et al. (2015) and Brown et al. (2024) were notable exceptions, with rigorous confounder management through ANCOVA-based verification and multilevel modeling, respectively. Sample representativeness was uncertain or inadequate in seven of eight studies, consistently reflecting convenience sampling from single schools or restricted geographic contexts.

The five qualitative studies demonstrated the most consistently adequate profiles overall. All five met the qualitative appropriateness criterion, four met methodological adequacy, data collection sufficiency, and findings representation criteria, and three met interpretive rigor criteria. Absent interrater reliability reporting for thematic coding affected three studies (Tan et al., 2019; Tan and Amiel, 2022; Hachem et al., 2022); Howard-Jones et al. (2015) used an informal analytic approach without a documented coding scheme. The four mixed methods studies showed adequate strand integration in three of four, with strand-specific limitations in Anderson et al. (2018) representing the primary methodological concern within this subgroup. The single descriptive study (Ribau, 2020) received the lowest overall rating, combining an un-validated instrument, absent inferential analysis, and a convenience sample without representativeness justification. Its findings are treated as illustrative rather than evidential throughout the synthesis.

Three methodological patterns recurred most consequentially across design types: absent or unverifiable outcome assessor blinding in most quantitative studies; inadequate fidelity reporting in one third of the corpus; and predominantly convenience-based sampling across all design types, with implications for geographic and socioeconomic representativeness. Table 4 synthesizes these patterns at the level of MMAT criteria across the full corpus.

**Table 4:** Cross-corpus MMAT compliance, by criterion (*N* = 24 studies).

MMAT criterion	Yes	Cannot tell	No	Pattern interpretation
Screening Q1: clear research questions	24	0	0	Universal compliance; minimum threshold met across the corpus
Screening Q2: data permit addressing questions	23	1	0	Universally adequate; single exception is the descriptive case study
C1: sample representativeness; randomization integrity	14	6	4	Convenience sampling dominates; randomization procedures often under-documented
C2: measurement appropriateness	20	1	3	Strongest area of the corpus; validated instruments used in the majority of studies
C3: complete outcome data; data collection adequacy	14	5	5	Attrition reporting and longitudinal data integrity are recurrent weaknesses
C4: confounder control; outcome assessor blinding; coherence	9	7	8	The most consistently problematic criterion; central to interpretive caution
C5: intervention fidelity; analytic-empirical alignment	16	8	0	Often reported, but fidelity documentation incomplete in one third of cases

Counts collapse design-specific C1–C5 labels (Hong et al., 2018) onto thematic categories sharing the same epistemic function across designs. Detailed per-design appraisals are available on OSF.

### 3.4 Results of individual sources of evidence

The relevant charted data for each of the 24 included studies are presented in Table 3, organized by study identifier and including country of origin, study design, educational level, theoretical foundations, translational process, researcher-educator collaboration level, and key outcomes. The complete charting matrix, encompassing all 17 extraction fields, is archived in the associated OSF project (see text footnote 3).

### 3.5 Synthesis of results

The synthesis follows the four-phase narrative procedure outlined by Popay et al. (2006), as described in the Methods section (2.5). Findings are organized in direct correspondence with the four review questions, with patterns qualified where they rest predominantly on studies with below-median MMAT scores. All synthesis materials are archived in the associated OSF project^5^.

#### 3.5.1 RQ1: theoretical principles guiding school-based EN studies

The theoretical landscape of the corpus was heterogeneous, with studies drawing on neuroscientific constructs at varying levels of specificity. The extent to which these constructs were explicitly linked to instructional design decisions varied substantially across the sample. Accordingly, the synthesis prioritized consistency with the conceptualizations adopted in the primary studies rather than imposing a unified classificatory framework. The clusters reported in this section reflect the constructs as the authors of the included studies labeled and operationalized them, rather than an externally imposed taxonomy. We acknowledge that, in influential current models (Diamond, 2013), attention is often treated as a component of executive functions, and that boundaries between adjacent constructs are conceptually contested. Authorial labeling is retained to preserve descriptive fidelity to the corpus, with overlap discussed explicitly where consequential for the synthesis.

Brain plasticity and neuroplasticity (Pascual-Leone et al., 2005; Lövdén et al., 2010) was the most frequently represented neuroscientific domain, invoked across nine studies as the foundational rationale for EN-informed interventions, including Hermida et al. (2015), Mackey et al. (2017), Anderson et al. (2018), Caballero and Llorent (2022), Skjermo et al. (2022), Brown et al. (2024), Koller (2024), and Zhumabayeva et al. (2025). In most of these cases, neuroplasticity functioned as a general principle supporting the claim that learning produces lasting neural change, rather than as an operationally specific construct informing design decisions. Executive functions and cognitive control (Miyake et al., 2000; Diamond, 2013) constituted the second most prevalent theoretical cluster, present across eight studies (Mackey et al., 2017; Letang et al., 2021; Zhumabayeva et al., 2025; Hachem et al., 2022; Runganurak et al., 2022; Allix et al., 2024; Caballero and Llorent, 2022; Hermida et al., 2015) and more consistently operationalized into concrete intervention components, particularly in studies targeting working memory, inhibitory control, and planning in primary school contexts. Attention (Petersen and Posner, 2012) appeared as a recurring theoretical reference across six studies (Khng and Mane, 2020; Letang et al., 2021; Roche-Cerasi et al., 2021; Massonnié et al., 2020; Runganurak et al., 2022; Skjermo et al., 2022), spanning classroom-level noise research and neuroimaging-informed test anxiety interventions, and was among the more directly operationalized constructs in the corpus. Emotion and motivation (Pessoa, 2008; Immordino-Yang and Damasio, 2007) were addressed in four studies (Howard-Jones et al., 2015; Elouafi et al., 2021; Brown et al., 2024; Koller, 2024), drawing on frameworks such as the neuro-evolutionary theory proposed by Panksepp and Biven (2012), the dopaminergic reward system articulated by Schultz (1998) and Fiorillo et al. (2003), as well as neurobiological models of the physiological stress response, particularly the HPA axis. These perspectives informed design decisions that departed from the predominantly cognitive emphasis observed across the rest of the sample.

A structurally important distinction emerged between studies drawing directly on primary neuroscience research and those grounding their theoretical rationale in secondary EN frameworks. Thirteen studies engaged with primary neuroscience sources and used them to derive specific instructional implications, including Diamond’s executive function research (Mackey et al., 2017; Letang et al., 2021; Hermida et al., 2015), Pulvermüller’s neural network hypothesis (Tan et al., 2019; Tan and Amiel, 2022), Panksepp’s affective neuroscience (Koller, 2024), the dopaminergic reward system as articulated by Schultz and Fiorillo (Howard-Jones et al., 2015), O’Keefe and Moser’s spatial navigation work (Roche-Cerasi et al., 2021; Skjermo et al., 2022), Westermann’s neuro-constructivism (Srikoon et al., 2024), neuroimaging evidence on EEG frequency bands (Khng and Mane, 2020), and McEwen’s allostatic load framework (Brown et al., 2024). Eleven studies drew predominantly on synthesized EN frameworks produced by scholars such as Tokuhama-Espinosa (Zhumabayeva et al., 2025; Allix et al., 2024; Ribau, 2020; Caballero and Llorent, 2022), Hardiman (Srikoon, 2019; Srikoon et al., 2024; Runganurak et al., 2022), and Masson (Bélanger et al., 2025), which aggregate neuroscientific evidence into principles or models already adapted for educational use. Studies drawing on primary neuroscience sources more consistently documented explicit chains of reasoning linking specific neuroscientific findings to specific instructional components. In contrast, studies relying on synthesized frameworks more often invoked neuroscience as legitimizing background, without making the underlying translational logic operationally explicit. This distinction is not a quality judgment on the studies concerned; rather, it reflects different translational positions each with different implications for the replicability and validation of the rationale underlying the study design.

A further recurring pattern across the corpus was the co-presence of neuroscientific and psychological theoretical frameworks. Studies rarely drew on neuroscience in isolation; rather, most situated EN constructs within broader cognitive, constructivist, or sociocultural traditions, including Vygotsky’s zone of proximal development (Zhumabayeva et al., 2025; Ribau, 2020), Bandura’s self-efficacy theory (Ribau, 2020; Elouafi et al., 2021), Dweck’s growth mindset model (Anderson et al., 2018; Koller, 2024), and self-determination theory (Elouafi et al., 2021; Roche-Cerasi et al., 2021). This theoretical pluralism is consistent with the interdisciplinary nature of EN as a field. It also reflects the practical necessity of connecting neuroscience to educational design traditions that teachers and curriculum developers already inhabit, a bridging function that the neuroscience literature alone does not yet fully provide.

#### 3.5.2 RQ2: translational processes and researcher-educator collaborations

The translational landscape of the corpus reflected substantial variation both in how neuroscientific knowledge was moved into school practice and in the degree to which educators participated in that process.

Using the T1–T4 translational framework as an analytical lens, the dominant translational position across the corpus was T2-T3, the implementation and evaluation of educational applications based on neuroscientific evidence, documented across 17 studies, including Hermida et al. (2015), Howard-Jones et al. (2015), Mackey et al. (2017), Anderson et al. (2018), Khng and Mane (2020), Massonnié et al. (2020), Chang et al. (2021), Elouafi et al. (2021), Letang et al. (2021), Roche-Cerasi et al. (2021), Caballero and Llorent (2022), Runganurak et al. (2022), Skjermo et al. (2022), Tan and Amiel (2022), Allix et al. (2024), and Zhumabayeva et al. (2025). These studies shared a common structural rationale: neuroscientific evidence or theory was used to design an educational intervention or professional development program, which was then implemented in authentic school settings and evaluated for its effects on learning, teaching, or neurophysiological outcomes. A smaller cluster of two studies focus on level T3 exclusively, examining the dissemination and implementation of neuroscience-informed knowledge within professional development communities without generating new primary evidence about classroom effects (Bélanger et al., 2025; Hachem et al., 2022). Three studies operated primarily at the T2 level, developing and testing instructional models without proceeding to broader dissemination (Srikoon, 2019; Koller, 2024; Srikoon et al., 2024). One study was positioned at the T1 translational stage, invoking neuroscientific principles of emotion and motivation as the explicit motivational rationale for the design and selection of the active laboratory station methodology, effectively bridging theoretical intent with pedagogical choice (Ribau, 2020). One study was positioned at the T1-T2 stage, with teaching applications framed on basic research using neurophysiological measurement to generate hypotheses about classroom-level variables rather than implementing a designed intervention (Brown et al., 2024). The T4 level, concerning the integration of EN-informed practices into broader educational systems and policies, was not represented as a primary translational objective in any study in the corpus.

No study was rated at the complete absence level; all four remaining categories were represented. Co-design was the most frequent designation (nine studies), followed by co-investigation (seven), implementation only (five), and consultation (three). Full study-level coding is presented in Table 3.

The depth and reciprocity of reported collaborations varied considerably within and across these categories. Among co-design studies, the most substantive examples involved iterative cycles of material development, classroom testing, and joint revision sustained over months or full academic years. This pattern was documented in Hermida et al. (2015), where weekly interdisciplinary meetings preceded implementation and ten-minute pre- and post-activity briefings maintained the feedback loop throughout the intervention; in Letang et al. (2021), where 2 months of preparatory co-construction via an online platform preceded data collection; in Howard-Jones et al. (2015), where five iterative cycles of design, intervention, analysis, and reflection across 4 years involved teachers in two schools as equal partners in shaping both technology design and pedagogy; and in Bélanger et al. (2025), where sessions of Professional Learning Communities (PLC), spread throughout the school year, enabled teachers to put their work into practice, reflect, and revise it between meetings. At the co-investigation level, Massonnié et al. (2020) exemplified this mode most fully, with the participating teacher functioning as a co-investigator from study inception, contributing to defining research questions, adapting measures, recruiting schools, and implementing the long-term intervention component. Tan et al. (2019), Tan and Amiel (2022), and Anderson et al. (2018) similarly engaged teachers as active agents of knowledge construction, with educators not merely implementing but interrogating, adapting, and eventually disseminating neuroscience-informed principles within their own professional communities. By contrast, implementation-focused studies such as Srikoon (2019), Khng and Mane (2020), and Srikoon et al. (2024) generally positioned educators as implementers of externally designed programs, with relatively limited reporting on their involvement in design or adaptation decisions.

A recurring structural feature across co-design and co-investigation studies was the presence of iterative feedback mechanisms, defined as documented cycles in which information from classroom implementation informed subsequent revision of intervention content or delivery. Such mechanisms were explicitly described in Hermida et al. (2015), Howard-Jones et al. (2015), Mackey et al. (2017), Anderson et al. (2018), Massonnié et al. (2020), Hachem et al. (2022), Caballero and Llorent (2022), Allix et al. (2024), and Bélanger et al. (2025). These mechanisms were documented in studies that also received higher collaboration ratings and presented more detailed reporting of implementation processes, suggesting a pattern of co-occurrence that warrants further investigation in primary research.

#### 3.5.3 RQ3: participant characteristics and research design

Participant characteristics influenced research design unevenly across the corpus. Sixteen studies focused primarily or exclusively on students, five on teachers or school personnel, and three on both (see Table 3 for the full mapping).

Among student-focused studies, the educational level of participants was the most consistently reported design-shaping variable. Studies targeting early childhood participants used shorter sessions, game-based formats, and simplified assessment tools consistent with preschool and kindergarten pedagogical traditions, as in Hermida et al. (2015) and Brown et al. (2024). Studies targeting secondary students employed more complex outcome batteries and longer intervention cycles. The developmental rationale for these choices was explicitly articulated in approximately half of the student-focused studies; in the remainder, educational level was reported as a contextual characteristic without documented implications for design.

Sample composition beyond educational level was inconsistently reported. Socioeconomic status was treated as a design consideration in three studies: Hermida et al. (2015) built an entire intervention around low-SES kindergarteners in Buenos Aires, incorporating socioeconomic and health-history variables as covariates and selecting tools validated for this population; Brown et al. (2024) recruited from Head Start preschools and included income-to-needs ratio as a covariate; Mackey et al. (2017) recruited predominantly low-income and Hispanic students and adapted group configuration accordingly. The presence of students with special educational needs or at-risk populations was acknowledged in a small number of studies, but only Mackey et al. (2017) and Hermida et al. (2015) documented how their inclusion shaped specific design decisions. Gender was reported as a demographic variable in most studies but rarely discussed as informing design choices.

Among teacher-focused studies, characteristics such as years of experience, subject area, prior neuroscience knowledge, and school context were consistently reported as contextual descriptors rather than as design variables. Voluntary participation introduced a recurring source of self-selection bias, with the sample skewed toward educators already predisposed toward EN; this limitation was explicitly noted by Hachem et al. (2022), Chang et al. (2021), and Tan et al. (2019). Setting itself was rarely treated as a design variable. Although thirteen studies were conducted in single-school contexts and eleven across multiple schools (see §3.2 and Table 3), only Massonnié et al. (2020) explicitly balanced school as a design factor, and only Hermida et al. (2015) used external control groups to address between-class and between-teacher variability.

#### 3.5.4 RQ4: conditions associated with the translation of neuroscientific knowledge into school practice

Across the corpus, three categories of conditions were documented as associated with the translation of neuroscientific knowledge into educational practice: barriers, facilitators, and implementation strategies. These are examined in turn, with findings qualified in relation to the methodological quality of the studies from which they are drawn.

The most frequently documented barrier to translation was curriculum overload and institutional time constraints, reported across multiple studies targeting teachers as participants, including Bélanger et al. (2025), Hachem et al. (2022), and Caballero and Llorent (2022). Educators consistently identified the competing demands of prescribed curricula, standardized assessment schedules, and administrative obligations as primary impediments to the adoption and sustained implementation of EN-informed practices. A related but distinct barrier was insufficient teacher preparation. Mackey et al. (2017), Srikoon (2019), Runganurak et al. (2022), and Hachem et al. (2022) reported that professional development of limited duration or intensity failed to produce the level of conceptual fluency required for confident and coherent classroom application; Runganurak et al. (2022) did not document teacher preparation in sufficient detail to support evaluation of this dimension. The language gap between neuroscientific terminology and educational discourse was identified as a structural barrier in Tan et al. (2019) and Tan and Amiel (2022), with teachers reporting difficulty internalizing neuroscience concepts without sustained researcher scaffolding. Neuromyth persistence emerged as a related challenge in the same two studies: even following EN-focused professional development, some teachers retained pre-existing misconceptions about learning and the brain that were not fully displaced by new knowledge.

The absence of formal fidelity measurement across 9 of the 24 studies, including Srikoon (2019), Massonnié et al. (2020), Elouafi et al. (2021), Letang et al. (2021), Runganurak et al. (2022), Skjermo et al. (2022), Caballero and Llorent (2022), Allix et al. (2024), and Zhumabayeva et al. (2025), limited confidence in conclusions about which specific translational practices were responsible for observed outcomes.

The conditions most consistently associated with successful translation across higher-quality studies converged around four structural features. Co-design and iterative collaboration sustained over extended time periods was associated with higher ecological validity and stronger teacher ownership of materials, as documented in Hermida et al. (2015), Howard-Jones et al. (2015), Letang et al. (2021), Massonnié et al. (2020), Allix et al. (2024), and Bélanger et al. (2025). Institutional and administrative support, including formal recognition of participation time, alignment with national curriculum standards, and involvement of school leadership in the research rationale, was identified as a critical enabling condition in Letang et al. (2021), Hachem et al. (2022), Roche-Cerasi et al. (2021), Skjermo et al. (2022). Embedding EN-informed practices within existing curriculum structures rather than creating supplementary sessions reduced implementation burden and increased sustainability, as reported in Letang et al. (2021), Caballero and Llorent (2022), and Hermida et al. (2015). The provision of accessible conceptual bridges between neuroscience and practice, whether through researcher-generated analogies in Tan et al. (2019) and Tan and Amiel (2022), structured discussion prompts in Koller (2024) and Bélanger et al. (2025), iterative low-fidelity prototyping with teachers as informant designers in Howard-Jones et al. (2015), or practitioner-authored training materials in Caballero and Llorent (2022), consistently supported teacher uptake of EN concepts without requiring scientific expertise.

A small subset of studies documented systematic monitoring of intervention delivery that helped resolve the interpretive uncertainty introduced by fidelity gaps elsewhere in the corpus. Hermida et al. (2015) deployed blind external observers using a structured classroom dynamic scale across fourteen activities per group, complemented by pre- and post-activity researcher-teacher briefings. Brown et al. (2024) coded child- and teacher-directed activities and teacher-child interaction quality from video using validated instruments with high interrater reliability. Massonnié et al. (2020) quantified within-group variability in protocol exposure as part of the analytic strategy itself, treating fidelity variance as a substantive finding rather than a methodological caveat. These cases indicate that documented monitoring is feasible within authentic school contexts, but remains the exception rather than the norm.

The most distinctive translational strategies documented in the corpus were those that operationalized feedback loops between classroom practice and intervention content. Studies that built structured mechanisms for teachers to report implementation experience back to researchers, and for that experience to inform subsequent revision of materials or delivery, achieved more coherent translation across the corpus, as documented in Hermida et al. (2015), Howard-Jones et al. (2015), Mackey et al. (2017), Anderson et al. (2018), Massonnié et al. (2020), Hachem et al. (2022), Caballero and Llorent (2022), Allix et al. (2024), and Bélanger et al. (2025). A second recurring strategy was the use of professional development structures combining theoretical content with immediate practical application, reflection, and peer discussion within the same session or intervention cycle. Professional learning communities (Bélanger et al., 2025), blended learning models (Anderson et al., 2018), and learning study cycles (Tan et al., 2019; Tan and Amiel, 2022) all instantiated variants of this approach and were associated with more documented evidence of transfer from Professional Development (PD) content to classroom practice. A third strategy, documented primarily in North American studies, was the engagement of teachers as disseminators of neuroscience knowledge within their own professional communities, creating secondary translational pathways that extended the reach of researcher-generated knowledge beyond the immediate study sample, as reported in Chang et al. (2021), Anderson et al. (2018), and Tan and Amiel (2022).

Effective translation, as documented across the corpus, appears to depend not on any single factor but on the interaction between structural conditions, collaborative processes, and iterative implementation mechanisms. The four thematic domains reported in Table 5 (institutional and structural conditions; teacher preparation and professional learning; conceptual mediation between neuroscience and educational discourse; implementation monitoring) were derived inductively from the included studies through reflexive thematic mapping conducted by both reviewers, then cross-checked against the T1–T4 translational framework to verify analytical coherence. The relationship between the inductive domains and the T-stages is not one-to-one: each domain operates across multiple T-stages, with implementation monitoring particularly active at T2-T3 and institutional conditions cutting across the entire framework. The thematic coding framework, including *a priori* categories, coding rules, and emergent themes, is documented on OSF (see text footnote 5). Individual pre-reconciliation coding files were not preserved in archivable form and are available from the corresponding author upon reasonable request. Entries in Table 5 are thematically grouped rather than paired one-to-one across columns. The implementation monitoring domain is asymmetrically populated, with documented barriers substantially outnumbering documented facilitators and strategies, reflecting an uneven distribution of methodological attention to monitoring across the corpus rather than a complete absence of such practices.

**Table 5:** Conditions associated with the translation of EN into school settings, organized by thematic domain.

Thematic domain	Barriers	Facilitators	Implementation strategies
Institutional and structural conditions	Curriculum overload and institutional time constraints (Bélanger et al., 2025; Hachem et al., 2022; Caballero and Llorent, 2022)	Institutional and administrative support (Letang et al., 2021; Hachem et al., 2022; Roche-Cerasi et al., 2021; Skjermo et al., 2022) Integration within existing curriculum structures (Letang et al., 2021; Caballero and Llorent, 2022; Hermida et al., 2015)	Iterative feedback loops between teachers and researchers embedded in collaborative design cycles (Mackey et al., 2017; Allix et al., 2024; Massonnié et al., 2020; Hermida et al., 2015; Bélanger et al., 2025; Hachem et al., 2022; Anderson et al., 2018; Caballero and Llorent, 2022)
Teacher preparation and professional learning	Insufficient teacher preparation and limited professional development opportunities (Mackey et al., 2017; Srikoon, 2019; Runganurak et al., 2022; Hachem et al., 2022)	Co-design and sustained researcher-educator collaboration over time (Hermida et al., 2015; Howard-Jones et al., 2015; Letang et al., 2021; Massonnié et al., 2020; Allix et al., 2024; Bélanger et al., 2025)	Professional development programs integrating theory, classroom practice, structured reflection, and peer discussion (Bélanger et al., 2025; Anderson et al., 2018; Tan et al., 2019; Tan and Amiel, 2022)
Conceptual mediation between neuroscience and educational discourse	Language gap between neuroscience and educational discourse (Tan et al., 2019; Tan and Amiel, 2022) Persistence of neuromyths despite training (Tan et al., 2019; Tan and Amiel, 2022)	Conceptual bridges translating neuroscientific findings into pedagogically accessible language (Howard-Jones et al., 2015; Tan et al., 2019; Tan and Amiel, 2022; Koller, 2024; Bélanger et al., 2025; Caballero and Llorent, 2022)	Engagement of teachers as disseminators of translated knowledge within professional communities (Chang et al., 2021; Anderson et al., 2018; Tan and Amiel, 2022)
Implementation monitoring	Absence of systematic fidelity measures, limiting the interpretability of implementation outcomes (Srikoon, 2019; Massonnié et al., 2020; Elouafi et al., 2021; Letang et al., 2021; Runganurak et al., 2022; Skjermo et al., 2022; Caballero and Llorent, 2022; Allix et al., 2024; Zhumabayeva et al., 2025)	Structured monitoring of intervention delivery through trained external observers and validated coding instruments (Hermida et al., 2015; Brown et al., 2024)	Pre- and post-activity researcher-teacher briefings and structured fidelity protocols (Hermida et al., 2015; Roche-Cerasi et al., 2021; Massonnié et al., 2020)

## 4 Discussion

### 4.1 Summary of evidence

The 24 included studies document an active field increasingly oriented toward applied contexts, but still characterized by significant conceptual and methodological heterogeneity. Progress in translating neuroscientific evidence into school settings is evident in the growing adoption of collaborative research designs and in the gradual identification of conditions that support evidence-based practice; however, persistent limitations remain in theoretical transparency, ecological representativeness, and systemic reach. For clarity the four translational stages are briefly recalled: T1 refers to the development of teaching applications based on basic research; T2 to their evaluation and synthesis into evidence-based guidelines; T3 to their implementation in practice; and T4 to their dissemination within educational systems and policies (Dresler et al., 2018; Fante, 2024). It should be noted that the corpus represents EN-self-identified research, retrieved through a search strategy anchored on the field’s canonical descriptors. Studies that engage substantively with translational questions but pitch themselves within cognitive psychology, cognitive training, or educational psychology vocabularies fall outside this synthesis. The patterns discussed below therefore describe the field as it identifies itself, rather than the broader domain of neuroscience-informed educational research (see Section 4.2).

Building on a broadly shared recognition that translating neuroscientific knowledge into educational practice is methodologically and conceptually demanding (Thomas et al., 2019; Morris and Sah, 2016), the corpus confirms this empirically. The concentration of studies in T2-T3 phases, the absence of formal fidelity monitoring in much of the corpus, and the persistence of neuromyths across the evidence constitute observable correlates of the disciplinary distance outlined in the rationale. Taken together, these patterns suggest that translation thus emerges not only as a conceptual challenge but as a practical condition shaping how EN research is designed, conducted, and reported.

The theoretical patterns identified in response to RQ1 reflect EN’s characteristic inter-disciplinarity. Constructs from cognitive neuroscience, particularly neuroplasticity, executive functions, and attention, dominated the corpus but were rarely applied in isolation: most studies situated these within broader psychological or educational frameworks, reflecting the practical necessity of connecting neuroscience to pedagogical traditions educators already inhabit. A smaller but notable subset drew on affective and motivational perspectives, including the neuro-evolutionary SEEKING framework derived from Panksepp’s work (Koller, 2024), neurophysiological models of stress regulation grounded in McEwen’s allostatic load theory (Brown et al., 2024), and motivational neuroscience anchored in self-determination theory and self-efficacy (Elouafi et al., 2021). These contributions suggest a gradual broadening of the field’s theoretical scope beyond its predominantly cognitive focus.

Beyond this broad interdisciplinary profile, a more specific pattern emerges, one that was anticipated in the rationale, and which has important implications for theoretical accountability 11 of the 24 studies drew primarily on synthesized EN frameworks without clearly tracing their translational logic back to primary neuroscientific evidence. This finding resonates with Weisberg et al.’s (2008) observation that references to neuroscience can enhance perceived credibility independently of explanatory depth, and it suggests that the boundary between research genuinely derived from neuroscientific findings and research merely framed in neuroscientific terms is more porous than often assumed In contrast, studies citing primary neuroscience sources—such as Diamond’s work on executive functions (Mackey et al., 2017; Letang et al., 2021; Hermida et al., 2015), Pulvermüller’s neural network hypotheses (Tan et al., 2019; Tan and Amiel, 2022), or McEwen’s allostatic load framework (Brown et al., 2024)—more consistently articulated explicit chains of reasoning linking neuroscientific evidence to instructional design decisions.

This distinction becomes particularly consequential in light of the T1–T4 translational framework underpinning the review. That framework was developed in genomic medicine to render the path from basic discovery to population-level application explicit and accountable at each stage (Khoury et al., 2007). When EN studies invoke theoretical frameworks without specifying where in this chain their claims originate, they effectively bypass the accountability logic the framework was designed to enforce. From this perspective, greater transparency regarding whether theoretical claims derive from primary neuroscience, synthesized models, or pedagogical inference would not only strengthen theoretical coherence but also enhance the interpretability and replicability of EN research.

A similar pattern of partial realization emerges when examining the translational configurations identified in response to RQ2. While T2-T3 translation clearly represents the dominant mode of school-based EN research during the period under review, the corpus demonstrates that studies occupying this space are far from homogeneous. They differ substantially in the extent to which neuroscientific evidence actively informs design decisions, as opposed to providing retrospective legitimation for pedagogical choices grounded in other traditions. Nevertheless, the overall pattern of collaboration documented in the corpus lends support to the transactional perspective articulated in the rationale, which conceptualizes translation as a dynamic and reciprocal process rather than a unidirectional transfer of knowledge from laboratory to classroom (Donoghue and Horvath, 2016; Tokuhama-Espinosa, 2010). The predominance of co-design and co-investigation models, along with the presence of iterative feedback mechanisms in the most coherent translational profiles, aligns with this perspective and reflects the non-linear character of the T1–T4 framework as originally theorized.

At the same time, the review also indicates that transactional principles are unevenly operationalized in practice. A subset of studies adopted implementation-only models (Srikoon, 2019; Khng and Mane, 2020; Runganurak et al., 2022; Srikoon et al., 2024; Zhumabayeva et al., 2025) and even within the co-design category the depth, reciprocity, and durability of collaboration varied considerably. While the transactional model is conceptually well established, it remains only partially realized in research practice. The trade-off between experimental control and ecological authenticity, observed across the corpus, is not a neutral methodological residue. Efficacy designs and authentic implementation studies answer different questions: the first establish whether an EN-informed intervention can produce a target outcome under controlled conditions; the second establish whether it does so when embedded in the constraints of routine schooling, with all the contextual variability that schooling entails (Cook and Campbell, 1986; Damschroder et al., 2009). Neither orientation is inherently superior. Greater experimental control protects against confounding and supports causal inference at T2, while authentic implementation protects against the artificial de-contextualization that has historically limited the transferability of laboratory findings to T3 practice. The argument that the holistic approach is intrinsically more realistic conflates two distinct claims: that authenticity is necessary for translational validity (which the corpus supports), and that authenticity is sufficient on its own (which it does not). Studies in the corpus that combined controlled comparison with ecological delivery, including Mackey et al. (2017), Letang et al. (2021), and Hermida et al. (2015), suggest that the two orientations can coexist within a single design rather than being mutually exclusive, provided that ecological constraints are treated as design parameters to be specified rather than as residual noise to be minimized. EN translation across the T1–T4 stages requires both kinds of evidence, and we do not claim that one is preferable. The corpus reviewed here is unevenly distributed across the trade-off, and this uneven distribution is itself a finding warranting interpretation rather than correction.

Heterogeneity in alignment with the T1–T4 framework can be read in two ways. As a descriptive feature of an emerging interdisciplinary field, it reflects the range of legitimate translational pathways available to researchers and educators. As an obstacle to cumulative knowledge building, it limits the possibility of comparing effect sizes, replicating implementations, and synthesizing findings across studies. Greater methodological standardization could deliver gains on the second front, but at the cost of premature closure, loss of contextual sensitivity, and tension with the multidirectional logic of T1–T4 itself. The present review treats heterogeneity as partly a marker of disciplinary immaturity and partly a constitutive feature of EN translation and argues that these two dimensions need to be kept analytically separate when planning future syntheses.

In line with these observations on heterogeneity, particularly in collaborative practices, more detailed reporting of how educator input concretely shapes intervention content, and of the conditions under which iterative feedback loops are sustained, would allow the field to move beyond aspirational accounts of collaboration toward cumulative knowledge about which forms of partnership support more coherent translation under specific conditions.

The limits of current translational practice become even more apparent when the systemic dimension of EN is considered. Drawing on Dresler et al.’s (2018) observation that the community and education component of translational EN has historically been underdeveloped, the present review finds strong empirical confirmation of this claim. None of the included studies treated systemic integration- such as influence on policy, curriculum frameworks, or institutional professional development structures- as a primary translational objective. As a result, a decade of applied EN research has generated relatively robust insight into the research-to-classroom interface, while leaving the classroom-to-system interface largely unexplored. This gap suggests that the potential for EN to inform educational systems, rather than isolated interventions, remains an open and largely unexamined question.

These systemic limitations are further compounded by the geographic concentration of the corpus. Studies conducted in low- or middle-income settings (Elouafi et al., 2021, Morocco; Hermida et al., 2015, Argentina; Runganurak et al., 2022 and Srikoon, 2019 and Srikoon et al., 2024, Thailand; Zhumabayeva et al., 2025, Kazakhstan) encountered qualitatively different conditions from those reported in high-income contexts, including constrained infrastructure, entrenched cultural practices, and structural pressures on curriculum and teacher time, suggesting that translational conditions identified in high-income contexts cannot be assumed to generalize without adaptation. Socioeconomic status was a design consideration in only three studies, and gender was rarely treated as a design variable in its own right, reflecting a broader tendency to treat context as a stable backdrop rather than as an active constraint on what forms of translation are feasible.

The conditions documented across the corpus converge on a recognizable pattern. Structural constraints, including curricular overload, limited training time, and persistent neuromyths, consistently impede translation, while facilitating conditions emerge when EN practices are embedded in existing curricula, supported institutionally, and developed through iterative co-design. Implementation strategies centered on feedback loops (documented in Hermida et al., 2015; Howard-Jones et al., 2015; Mackey et al., 2017; Anderson et al., 2018; Massonnié et al., 2020; Hachem et al., 2022; Caballero and Llorent, 2022; Allix et al., 2024; Bélanger et al., 2025), integrated professional development (documented in Anderson et al., 2018; Tan et al., 2019; Tan and Amiel, 2022; Bélanger et al., 2025), and teachers as active knowledge mediators (documented in Anderson et al., 2018; Chang et al., 2021; Tan and Amiel, 2022) were consistently associated with more coherent translational outcomes.

The three gaps identified in the review rationale as motivating the study acquire greater epistemic weight. The heterogeneity of theoretical foundations reflects unresolved disagreement within the field about what constitutes adequate neuroscientific grounding and where accountability for theoretical claims should reside along the translational chain. The inconsistent documentation of researcher-educator collaboration obscures the mechanisms through which practitioner expertise shapes both intervention quality and sustainability. Finally, the weak integration of participant characteristics into design decisions reveals a systematic tendency to under-theorize context. Addressing these issues will require not only improved reporting standards, but a fundamental reorientation in how EN research defines its theoretical accountability, its collaborative practices, and its engagement with educational contexts.

### 4.2 Limitations

Some limitations should be acknowledged. The most consequential methodological deviation from JBI guidance concerned the screening procedure: although calibration yielded high inter-rater agreement (Cohen’s kappa = 0.807), title and abstract screening of the remaining records was divided between reviewers rather than conducted independently across the full corpus. This decision, adopted on practical grounds, was mitigated through conservative decision rules and systematic cross-checking, but the possibility that eligible studies were excluded by a single reviewer cannot be entirely ruled out. A similar constraint applied to data charting, where extraction was shared and cross-comparison of completed matrices served as the primary reliability safeguard.

The restriction to English-language publications and exclusion of gray literature further limit the synthesis. The English-only criterion likely underrepresents work from non-Anglophone contexts, and the exclusion of practitioner reports and institutional evaluations may have omitted ecologically mature translational work outside academic publication channels. Both constraints compound the geographic concentration of the corpus.

A further constraint arises from the lexical architecture of the search strategy itself. The Boolean strings privileged field self-identification, requiring at least one of the canonical field descriptors (educational neuroscience, neuroeducation, neuro-education, neuro-educational, mind, brain, and education, mind brain education) in title, abstract or keywords. This design choice ensured precision and corpus coherence but rendered invisible a body of work that is grounded in cognitive neuroscience, engages substantively with translational questions, and informs pedagogical design without adopting the field’s self-identifying lexicon. Studies pitched primarily within cognitive psychology, cognitive training, or educational psychology vocabularies, even when they cite EN frameworks, draw on neuroimaging evidence, and embed cognitive control training within curricular content, fall outside the retrievable corpus. The boundaries of the field as reviewed here are therefore defined by author self-identification as much as by translational substance, and the corpus should be read as representing EN-self-identified research rather than the broader space of neuroscience-informed educational interventions.

The relatively small corpus of 24 studies reflects the stringent operationalization of eligibility criteria, particularly the requirement that studies document the implementation of a neuroscience-informed educational action in an authentic school context. This limits the statistical weight of frequency-based conclusions but enhances analytical coherence.

The use of the T1–T4 framework, developed in genomic medicine (Khoury et al., 2007) and subsequently adapted to EN (Dresler et al., 2018; Fante, 2024), inevitably foregrounds certain dimensions of translation while rendering others less visible. Alternative frameworks for conceptualizing educational knowledge translation (Donoghue and Horvath, 2016; Coch and Daniel, 2025) might have produced a differently configured synthesis. Finally, the widespread underreporting of intervention fidelity in primary studies admits two distinct interpretations that are worth keeping separate. The first is a matter of reporting practice: limited fidelity-reporting conventions in the EN literature, partly attributable to journal expectations, leave the reader unable to reconstruct the operative components of an intervention as delivered. The second is more substantive: there is genuine theoretical and methodological disagreement in implementation studies of complex educational interventions about what fidelity should mean, with strict adherence to a manualized protocol potentially in tension with the adaptive, context-sensitive practice that authentic classroom translation requires. Part of the heterogeneity in the corpus reflects substantive debate, not only differences in reporting completeness, and the limitation should be read accordingly. This represents a limitation of the available evidence rather than of the review process itself.

## 5 Conclusion

EN-informed research conducted in authentic school contexts has progressed substantially over the past decade, with the corpus documenting collaborative designs, rigorous measurement approaches, and ecologically grounded implementation. Taken together, these findings suggest that the central challenge of EN translation lies not in the absence of applications, but in the conditions under which translation becomes theoretically accountable, methodologically coherent, and scalable across educational contexts. Notably, no study in the corpus addressed T4-level translation, indicating sustained attention to the *research-to-classroom* interface alongside a persistent neglect of the *classroom-to-system* interface. Addressing this gap will require research that takes institutional adoption, teacher education reform, and policy integration as primary empirical objects rather than as downstream implications.

For researchers, the evidence points to the need for greater transparency in the chain of reasoning linking specific neuroscientific findings to instructional design, including explicit specification of the translational stage at which theoretical claims operate. More systematic documentation of researcher-educator collaboration and wider adoption of formal fidelity monitoring would strengthen replicability and interpretability of outcomes. Studies in the corpus that operationalized systematic monitoring of intervention delivery (Hermida et al., 2015; Massonnié et al., 2020; Brown et al., 2024) demonstrate that this is feasible within authentic school contexts and offer concrete templates that future research can adapt.

For practitioners and professional development designers, the evidence favors sustained, iterative engagement with researchers over short-term, unidirectional training models. Professional learning communities (as exemplified by Bélanger et al., 2025), learning study cycles (Tan et al., 2019; Tan and Amiel, 2022), and blended professional development formats (Anderson et al., 2018; Hachem et al., 2022) appear promising structures for this purpose, with documented evidence of transfer from PD content to classroom practice in the studies cited above, though their effectiveness in EN contexts specifically remains to be examined more systematically.

At the policy level, recurring structural barriers, including curriculum overload, time constraints, and absent formal recognition for research participation, are systemic in nature and require systemic responses: protected time for collaboration, alignment of professional development structures with sustained engagement, and institutional recognition of researcher-educator partnerships as legitimate professional activity.

A decade of school-based EN research has produced encouraging evidence that neuroscience-informed practices can be implemented in authentic classrooms when supported by sustained collaboration, institutional alignment, and methodological transparency. The next decade will need to test whether this evidence base can scale beyond individual interventions to inform the systems within which schools operate.

## Footnotes

1
folder 02. Search

2
folder 03. Screening

3
folder 04. Data charting

4
folder 05. Quality assessment

5
folder 06. Synthesis

## References

[ref1] Allix P. Lubin A. Lanoë C. Mortier A. Rossi S. (2024). Impact of the metacognitive educational program Cogni’Scol on the academic success of middle school students. Mind Brain Educ. 18, 173–186. doi: 10.1111/mbe.12398

[ref2] Anderson R. K. Boaler J. Dieckmann J. A. (2018). Achieving elusive teacher change through challenging myths about learning: a blended approach. Educ. Sci. 8:98. doi: 10.3390/educsci8030098

[ref3] Ansari D. Coch D. (2006). Bridges over troubled waters: education and cognitive neuroscience. Trends Cogn. Sci. 10, 146–151. doi: 10.1016/j.tics.2006.02.007, 16530462

[ref4] Ansari D. De Smedt B. Grabner R. H. (2012). Neuroeducation-a critical overview of an emerging field. Neuroethics 5, 105–117. doi: 10.1007/s12152-011-9119-3

[ref5] Arksey H. O’Malley L. (2005). Scoping studies: towards a methodological framework. Int. J. Soc. Res. Methodol. 8, 19–32. doi: 10.1080/1364557032000119616

[ref6] Aromataris E. Lockwood C. Munn Z. Porritt K. Stern C. (2024). JBI Manual for Evidence Synthesis. Adelaide: JBI.

[ref7] Bélanger É. McMullin S. Hould E. Brault Foisy L. M. Masson S. (2025). Factors that facilitate or impede the implementation of neuroeducational principles: perspectives from preschool and primary school teachers. Mind Brain Educ. 19, 83–89. doi: 10.1111/mbe.70004

[ref8] Brown E. D. Holochwost S. J. Wolf D. P. Allen A. A. Garnett M. L. Velazquez-Martin B. . (2024). Music education and neurophysiological regulation in early childhood: should teachers guide or get out of the way? Mind Brain Educ. 18, 360–372. doi: 10.1111/mbe.12370

[ref9] Caballero M. Llorent V. J. (2022). The effects of a teacher training program on neuroeducation in improving reading, mathematical, social, emotional and moral competencies of secondary school students. A two-year quasi-experimental study. Revista. Psicodidáctica. 27, 158–167. doi: 10.1016/j.psicoe.2022.04.002

[ref10] Chang Z. Schwartz M. S. Hinesley V. Dubinsky J. M. (2021). Neuroscience concepts changed teachers’ views of pedagogy and students. Front. Psychol. 12:685856. doi: 10.3389/fpsyg.2021.685856, 34456800 PMC8384951

[ref11] Christodoulou J. A. Gaab N. (2009). Using and misusing neuroscience in education-related research. Cortex 45, 555–557. doi: 10.1016/j.cortex.2008.06.004, 18644589

[ref12] Coch D. Daniel D. B. (2025). Connecting Neuroscience with Education: Critical Considerations. Leiden: Brill.

[ref13] Cook T. D. Campbell D. T. (1986). The causal assumptions of quasi-experimental practice: the origins of quasi-experimental practice. Synthese 68, 141–180. doi: 10.1007/BF00413970

[ref14] Damschroder L. J. Aron D. C. Keith R. E. Kirsh S. R. Alexander J. A. Lowery J. C. (2009). Fostering implementation of health services research findings into practice: a consolidated framework for advancing implementation science. Implement. Sci. 4:50. doi: 10.1186/1748-5908-4-50, 19664226 PMC2736161

[ref15] Diamond A. (2013). Executive functions. Annu. Rev. Psychol. 64, 135–168. doi: 10.1146/annurev-psych-113011-143750, 23020641 PMC4084861

[ref16] Donoghue G. M. Horvath J. C. (2016). Translating neuroscience, psychology and education: an abstracted conceptual framework for the learning sciences. Cogent Educ. 3:1267422. doi: 10.1080/2331186X.2016.1267422

[ref17] Dresler T. Bugden S. Gouet C. Lallier M. Oliveira D. G. Pinheiro-Chagas P. . (2018). A translational framework of educational neuroscience in learning disorders. Front. Integr. Neurosci. 12:25. doi: 10.3389/fnint.2018.00025, 30022931 PMC6039789

[ref18] Edelenbosch R. Kupper F. Krabbendam L. Broerse J. E. (2015). Brain-based learning and educational neuroscience: boundary work. Mind Brain Educ. 9, 40–49. doi: 10.1111/mbe.12066

[ref19] Elouafi L. Lotfi S. Talbi M. (2021). Progress report in neuroscience and education: experiment of four neuropedagogical methods. Educ. Sci. 11:373. doi: 10.3390/educsci11080373

[ref20] Fante C. (2024) Educational neuroscience and teacher neuro-literacy: a translational and action framework for teacher training ICERI 2024 Proceedings (pp. 9702–9708) Valencia: IATED

[ref21] Feiler J. B. Stabio M. E. (2018). Three pillars of educational neuroscience from three decades of literature. Trends Neurosci. Educ. 13, 17–25. doi: 10.1016/j.tine.2018.11.001

[ref22] Fiorillo C. D. Tobler P. N. Schultz W. (2003). Discrete coding of reward probability and uncertainty by dopamine neurons. Science 299, 1898–1902. doi: 10.1126/science.1077349, 12649484

[ref23] Fischer K. W. Goswami U. Geake J. Task Force on the Future of Educational Neuroscience (2010). The future of educational neuroscience. Mind Brain Educ. 4, 68–80. doi: 10.1111/j.1751-228X.2010.01086.x

[ref24] Goswami U. (2004). Neuroscience, education and special education. Br. J. Spec. Educ. 31, 175–183. doi: 10.1111/j.0952-3383.2004.00352.x

[ref25] Hachem M. Daignault K. Wilcox G. (2022). Impact of educational neuroscience teacher professional development: perceptions of school personnel. Front. Educ. 7:912827. doi: 10.3389/feduc.2022.912827

[ref26] Han H. Soylu F. Anchan D. M. (2019). Connecting levels of analysis in educational neuroscience: a review of multi-level structure of educational neuroscience with concrete examples. Trend. Neurosci. Educ. 17:100113. doi: 10.1016/j.tine.2019.100113, 31685129

[ref27] Hermida M. J. Segretin M. S. Prats L. M. Fracchia C. S. Colombo J. A. Lipina S. J. (2015). Cognitive neuroscience, developmental psychology, and education: interdisciplinary development of an intervention for low socioeconomic status kindergarten children. Trends Neurosci. Educ. 4, 15–25. doi: 10.1016/j.tine.2015.03.003

[ref28] Hong Q. N. Fàbregues S. Bartlett G. Boardman F. Cargo M. Dagenais P. . (2018). The mixed methods appraisal tool (MMAT) version 2018 for information professionals and researchers. Educ. Inf. 34, 285–291. doi: 10.3233/EFI-180221

[ref29] Howard-Jones P. (2007). Neuroscience and Education: Issues and Opportunities. London: TLRP Commentary.

[ref30] Howard-Jones P. A. (2014). Neuroscience and education: myths and messages. Nat. Rev. Neurosci. 15, 817–824. doi: 10.1038/nrn3817, 25315391

[ref31] Howard-Jones P. Holmes W. (2017). “Neuroscience research and classroom practice,” in From the Laboratory to the Classroom: Translating Science of Learning for Teachers, eds. Horvath J. C. Lodge J. M. Hattie J. (London: Routledge/Taylor & Francis Group), 253–278.

[ref32] Howard-Jones P. Holmes W. Demetriou S. Jones C. Tanimoto E. Morgan O. . (2015). Neuroeducational research in the design and use of a learning technology. Learn. Media Technol. 40, 227–246. doi: 10.1080/17439884.2014.943237

[ref33] Immordino-Yang M. H. Damasio A. (2007). We feel, therefore we learn: the relevance of affective and social neuroscience to education. Mind Brain Educ. 1, 3–10. doi: 10.1111/j.1751-228X.2007.00004.x

[ref34] Keramaris N. C. Kanakaris N. K. Tzioupis C. Kontakis G. Giannoudis P. V. (2008). Translational research: from benchside to bedside. Injury 39, 643–650. doi: 10.1016/j.injury.2008.01.051, 18508055

[ref35] Khng K. H. Mane R. (2020). Beyond BCI—validating a wireless, consumer-grade EEG headset against a medical-grade system for evaluating EEG effects of a test anxiety intervention in school. Adv. Eng. Inform. 45:101106. doi: 10.1016/j.aei.2020.101106

[ref36] Khoury M. J. Gwinn M. Yoon P. W. Dowling N. Moore C. A. Bradley L. (2007). The continuum of translation research in genomic medicine: how can we accelerate the appropriate integration of human genome discoveries into health care and disease prevention? Genet. Med. 9, 665–674. doi: 10.1097/GIM.0b013e31815699d0, 18073579

[ref37] Koller K. (2024). Integrating exploration as a learning context impacts feelings of empowerment and engagement. Int. J. Educ. Res. Open 7:100374. doi: 10.1016/j.ijedro.2024.100374

[ref38] Legrenzi P. Umiltà C. (2011). Neuromania: On the Limits of brain Science. Oxford: Oxford University Press.

[ref39] Letang M. Citron P. Garbarg-Chenon J. Houdé O. Borst G. (2021). Bridging the gap between the lab and the classroom: an online citizen scientific research project with teachers aiming at improving inhibitory control of school-age children. Mind Brain Educ. 15, 122–128. doi: 10.1111/mbe.12272

[ref40] Lövdén M. Bodammer N. C. Kühn S. Kaufmann J. Schütze H. Tempelmann C. . (2010). Experience-dependent plasticity of white-matter microstructure extends into old age. Neuropsychologia 48, 3878–3883. doi: 10.1016/j.neuropsychologia.2010.08.026, 20816877

[ref41] Mackey A. P. Park A. T. Robinson S. T. Gabrieli J. D. (2017). A pilot study of classroom-based cognitive skill instruction: effects on cognition and academic performance. Mind Brain Educ. 11, 85–95. doi: 10.1111/mbe.12138

[ref42] Massonnié J. Frasseto P. Mareschal D. Kirkham N. Z. (2020). Scientific collaboration with educators: practical insights from an in-class noise-reduction intervention. Mind Brain Educ. 14, 303–316. doi: 10.1111/mbe.12240

[ref43] Miyake A. Friedman N. P. Emerson M. J. Witzki A. H. Howerter A. Wager T. D. (2000). The unity and diversity of executive functions and their contributions to complex “frontal lobe” tasks: a latent variable analysis. Cogn. Psychol. 41, 49–100. doi: 10.1006/cogp.1999.0734, 10945922

[ref44] Morris J. Sah P. (2016). Neuroscience and education: mind the gap. Aust. J. Educ. 60, 146–156. doi: 10.1177/0004944116652913

[ref45] Panksepp J. Biven L. (2012). “A meditation on the affective neuroscientific view of human and animalian MindBrains,” in From the Couch to the Lab: Trends in Psychodynamic Neuroscience, eds. Fotopoulou A. Pfaff D. Conway M. A. (Oxford: Oxford University Press), 145–175.

[ref46] Pascual-Leone A. Amedi A. Fregni F. Merabet L. B. (2005). The plastic human brain cortex. Annu. Rev. Neurosci. 28, 377–401. doi: 10.1146/annurev.neuro.27.070203.144216, 16022601

[ref47] Pessoa L. (2008). On the relationship between emotion and cognition. Nat. Rev. Neurosci. 9, 148–158. doi: 10.1038/nrn2317, 18209732

[ref48] Peters M. D. Marnie C. Tricco A. C. Pollock D. Munn Z. Alexander L. . (2020). Updated methodological guidance for the conduct of scoping reviews. JBI Evid. Synth. 18, 2119–2126. doi: 10.11124/JBIES-20-00167, 33038124

[ref49] Petersen S. E. Posner M. I. (2012). The attention system of the human brain: 20 years after. Annu. Rev. Neurosci. 35, 73–89. doi: 10.1146/annurev-neuro-062111-150525, 22524787 PMC3413263

[ref50] Pollock D. Peters M. D. Khalil H. McInerney P. Alexander L. Tricco A. C. . (2023). Recommendations for the extraction, analysis, and presentation of results in scoping reviews. JBI Evid. Synth. 21, 520–532. doi: 10.11124/JBIES-22-00123 36081365

[ref51] Popay J. Roberts H. Sowden A. Petticrew M. Arai L. Rodgers M. . (2006). Guidance on the Conduct of Narrative Synthesis in Systematic Reviews: A Product from the ESRC Methods Programme. Lancaster: Lancaster University.

[ref52] Privitera A. J. (2021). A scoping review of research on neuroscience training for teachers. Trends. Neurosci. Educ. 24:100157. doi: 10.1016/j.tine.2021.100157, 34412863

[ref53] Privitera A. J. Ng S. H. S. Chen S. H. A. (2023). Defining the science of learning: a scoping review. Trends. Neurosci. Educ. 32:100206. doi: 10.1016/j.tine.2023.100206, 37689432

[ref54] Ribau I. (2020). Practical work by laboratory stations: an innovation in experimental work. Univ. J. Educ. Res. 8, 17–26. doi: 10.13189/ujer.2020.080103

[ref55] Roche-Cerasi I. Moe D. Skjermo J. Wigum J. P. (2021) Innovative road safety education program. In: Proceedings of the 31st European Safety and Reliability Conference 10 978–981 Trondheim: Norwegian Research Information Repository

[ref56] Runganurak W. Bunterm T. Uopasai S. Tang K. N. (2022). The effect of design-based learning integrated with educational neuroscience instructional model on students’ learning outcomes, executive functions, and learning stress. Pertanika J. Soc. Sci. Humanit. 30, 813–834. doi: 10.47836/pjssh.30.2.21

[ref57] Schultz W. (1998). Predictive reward signal of dopamine neurons. J. Neurophysiol. 80, 1–27. doi: 10.1152/jn.1998.80.1.1, 9658025

[ref58] Skjermo J. Roche-Cerasi I. Moe D. Opland R. (2022). Evaluation of road safety education program with virtual reality eye tracking. SN Comput. Sci. 3:149. doi: 10.1007/s42979-022-01036-w

[ref59] Srikoon S. (2019). Effect of 5P model on mathematics achievement and mood. J. Phys. Conf. Ser. 1340:012021. doi: 10.1088/1742-6596/1340/1/012021

[ref60] Srikoon S. Khamput C. Punsrigate K. (2024). Effects of stemen teaching models on mathematical literacy and mathematical problem-solving. Malays. J. Learn. Instr. 21, 79–115. doi: 10.32890/mjli2024.21.2.4

[ref61] Szűcs D. Goswami U. (2007). Educational neuroscience: defining a new discipline for the study of mental representations. Mind Brain Educ. 1, 114–127. doi: 10.1111/j.1751-228X.2007.00012.x

[ref62] Tan Y. S. M. Amiel J. J. (2022). Teachers learning to apply neuroscience to classroom instruction: case of professional development in British Columbia. Prof. Dev. Educ. 48, 70–87. doi: 10.1080/19415257.2019.1689522

[ref63] Tan Y. S. M. Amiel J. J. Yaro K. (2019). Developing theoretical coherence in teaching and learning: case of neuroscience-framed learning study. Int. J. Lesson Learn. Stud. 8, 229–243. doi: 10.1108/IJLLS-10-2018-0072

[ref64] Thomas M. S. Ansari D. Knowland V. C. (2019). Annual research review: educational neuroscience: progress and prospects. J. Child Psychol. Psychiatry 60, 477–492. doi: 10.1111/jcpp.12973, 30345518 PMC6487963

[ref65] Thomas M. S. C. Arslan Y. (2025). Why does the brain matter for education? Br. J. Educ. Psychol. 95, 303–320. doi: 10.1111/bjep.12727, 39630154 PMC12068028

[ref66] Tokuhama-Espinosa T. (2010). Mind, Brain, and Education Science: A Comprehensive Guide to the New Brain-Based Teaching. New York, NY: WW Norton & Company.

[ref67] Tokuhama-Espinosa T. Nouri A. (2023). Teachers’ mind, brain, and education literacy: a survey of scientists’ views. Mind Brain Educ. 17, 170–174. doi: 10.1111/mbe.12377

[ref68] Tommerdahl J. (2010). A model for bridging the gap between neuroscience and education. Oxford Rev. Educ. 36, 97–109. doi: 10.1080/03054980903518936

[ref69] Tricco A. C. Lillie E. Zarin W. O’Brien K. K. Colquhoun H. Kastner M. . (2016). A scoping review on the conduct and reporting of scoping reviews. BMC Med. Res. Methodol. 16, 1–10. doi: 10.1186/s12874-016-0116-4, 26857112 PMC4746911

[ref70] Tricco A. C. Lillie E. Zarin W. O’Brien K. K. Colquhoun H. Levac D. . (2018). PRISMA extension for scoping reviews (PRISMA-ScR): checklist and explanation. Ann. Intern. Med. 169, 467–473. doi: 10.7326/M18-0850, 30178033

[ref71] Weisberg D. S. Keil F. C. Goodstein J. Rawson E. Gray J. R. (2008). The seductive allure of neuroscience explanations. J. Cogn. Neurosci. 20, 470–477. doi: 10.1162/jocn.2008.20040, 18004955 PMC2778755

[ref72] Zhumabayeva Z. Bazarbekova R. Nurzhanova S. Stambekova A. Kalbergenova S. B. (2025). Development of neuro-didactic content aimed at developing the intelligence of younger schoolchildren. Front. Educ. 10:1584490. doi: 10.3389/feduc.2025.1584490

